# Real-World Safety of Cyproheptadine-Based Appetite Stimulants: An Electronic Health Record-Based Retrospective Cohort Study in Adult Patients

**DOI:** 10.3390/jcm15010054

**Published:** 2025-12-21

**Authors:** Minoh Ko, Kwangsoo Kim, Heeman Jang, Soomin Lee, Bumkyu Shin, Belong Cho, Seungyeon Kim, Ha Young Jang

**Affiliations:** 1 College of Pharmacy, Daegu Catholic University, Gyeongsan 38430, Republic of Korea; moko@cu.ac.kr; 2 Division of Clinical Bioinformatics, Biomedical Research Institute, Seoul National University Hospital, Seoul 03080, Republic of Korea; 3 Samjin Pharmaceutical Co., Ltd., Seoul 04054, Republic of Korea; 4 Department of Family Medicine, Seoul National University Hospital, Seoul National University College of Medicine, Seoul 07061, Republic of Korea; 5 College of Pharmacy, Dankook University, Cheonan 31116, Republic of Korea; 6 College of Pharmacy, Gachon University, Incheon 13120, Republic of Korea

**Keywords:** cyproheptadine-based appetite stimulants, real-world data, common data model, safety, dizziness, sedation, hypotension

## Abstract

**Background**: Cyproheptadine-based appetite stimulants (CAS) have been safely used in Korea for over 30 years. However, in older adults who are vulnerable to malnutrition, sarcopenia, and fall-related morbidity, safety of CAS in nutrition care remains uncertain due to limited evidence and its antihistaminic effects. This study aimed to assess the real-world safety of CAS compared with megestrol and other antihistamines to inform safe pharmacologic support within clinical nutrition practice. **Methods**: A retrospective observational study was conducted using Seoul National University Hospital’s common data model. Patients who were prescribed CAS, megestrol, or antihistamines between 2004 and 2022 were enrolled. To balance covariates, propensity score matching was applied. The primary outcomes—dizziness, sedation, and hypotension—were evaluated within 30 days of drug administration. Additionally, sensitivity analyses and subgroup assessments by age and duration of use were performed to evaluate robustness of the findings. **Results**: No significant differences were observed in the risk of dizziness, sedation, or hypotension when CAS was compared to megestrol, with adjusted hazard ratios (aHRs) and 95% confidence intervals (CIs) of 1.02 (0.70–1.50) for dizziness, 0.53 (0.19–1.54) for sedation, and 0.70 (0.34–1.44) for hypotension. Similar findings were noted in the comparison with antihistamines, where the aHRs for dizziness, sedation, or hypotension of 0.56 (0.41–0.78), 1.05 (0.46–2.38), and 0.65 (0.36–1.17), respectively. **Conclusions**: CAS demonstrated an acceptable safety profile in older adults, with safety comparable to both megestrol and antihistamines.

## 1. Introduction

Malnutrition is a common problem across the adult population and is associated with adverse health outcomes, including impaired functional status, increased morbidity, and reduced quality of life [1]. As nutritional deficits progress, appetite suppression and catabolic signaling can lead to clinically significant anorexia, and the consequences are particularly pronounced in older adults who are more vulnerable to sarcopenia, disability, and functional decline [2]. Pooled estimates from multiple countries indicate that its prevalence ranges from 8.5% in community settings to 28.0% in hospital settings, based on assessments using 22 validated malnutrition screening tools [3]. When anorexia is not addressed in a timely manner, it can precipitate or worsen geriatric syndromes, exacerbating sarcopenia, disability, functional impairment, and cachexia.

Cyproheptadine-based appetite stimulants (CAS), containing cyproheptadine orotate along with cyanocobalamin, carnitine hydrochloride, and lysine hydrochloride, have been widely prescribed in Korea as an appetite stimulant for over 30 years in clinical nutrition practice. Its efficacy and tolerability have been reported in randomized clinical trials and corroborated by extensive clinical use [4,5]. Cyproheptadine acts as a 5-HT2 receptor antagonist, blocking the appetite-suppressing effects of serotonin and thereby promoting appetite and potentially increasing energy intake. It also functions as a histamine 1 receptor antagonist, creating challenges for its use among elderly or debilitated patients. Notably, in the U.S., first-generation antihistamines are known to increase the risks of dizziness, sedation, and hypotension among the elderly. Consequently, warnings advise against engaging in activities that require mental alertness, and these agents are contraindicated in debilitated elderly patients [6]. However, the cyproheptadine dosage used in CAS (6 mg/day) is considerably lower than that typically indicated for its antihistaminic effects under the U.S. guidelines (recommended: 4–20 mg/day; maximum: 32 mg/day). Moreover, other antihistamine-containing drugs approved in Korea do not carry specific contraindications for elderly patients. Additionally, in Spain, where cyproheptadine was approved as an appetite stimulant, such warnings are not present [7]. These differing regulatory contexts raise questions about whether CAS presents a higher risk of dizziness, sedation, or hypotension compared to other appetite stimulants or antihistamines.

The efficacy and tolerability of cyproheptadine for poor appetite were evaluated in randomized double-blind placebo-controlled study [8]. The results demonstrated statistically significant improvements in weight and body mass index, with tolerable side effects such as somnolence. However, this study population was limited to adults aged 19 to 64 years with poor appetite. Megestrol, another option for appetite control, presents safety concerns including thromboembolic events or metabolic syndromes, and is indicated mainly for cancer or AIDS patients [9,10]. These limitations leave a gap in effective options for managing anorexia in elderly patients, highlighting the potential need for cyproheptadine in this population supported by robust scientific evidence on safety. In such scenarios, when imminent clinical evaluation and reliable real-world evidence are needed, The Observational Medical Outcomes Partnership (OMOP) Common data model (CDM) can be invaluable [11]. CDM uses data from electronic health records (EHRs), standardized into structured concepts, which allows for population-level effect estimation or patient-level prediction studies while maintaining individual patient privacy.

Given the clinical use of CAS as an appetite stimulant, particularly among older adults, further safety evaluations are crucial. This study addresses this gap by retrospectively analyzing two decades of clinical data to assess the safety of CAS in adult patients, including older adults, comparing it with alternative appetite stimulants, megestrol and other commonly used antihistamines.

## 2. Materials and Methods

### 2.1. Study Design and Data Sources

This was a retrospective, observational study that utilized EHRs standardized to the OMOP CDM version 5.4, supported by the Observational Health Data Sciences and Informatics (OHDSI) open-source platform. The data were sourced from Seoul National University Hospital (SNUH), comprising de-identified EHRs from approximately six million patients between 2004 and 2023. Medications recorded in the SNUH-CDM (SCDM) were mapped to RxNorm and RxNorm Extension concept identifiers (IDs) based on drug name, dosage, and administration details. Diagnoses were aligned by mapping internal diagnosis codes to corresponding SNOMED CT concept IDs through text matching. Similarly, laboratory tests were standardized using LOINC Vocabulary concept IDs, with specimen type and unit information serving as matching criteria. Procedures and surgeries were likewise mapped to SNOMED CT Vocabulary concept IDs, ensuring a comprehensive and uniform dataset for the subsequent analyses.

### 2.2. Patient Selection and Follow-Up

Patients who received at least one dose of CAS, megestrol or other antihistamines between 2004 and 2022 were eligible for inclusion. Patients were excluded if they were younger than 18 years old or had a prior history of outcomes of interest. The eligible cohort was assigned to one of three groups based on the first medication used: CAS, megestrol (control group 1), or other antihistamines (control group 2). The enrollment date for each patient was the first day of medication use. For the primary analysis, time-at-risk began on the index date and continued until 30 days after the last administration of the index medication. Patients were censored at the earliest occurrence of death, loss to follow-up, or switching to a comparator medication; specifically, CAS users who subsequently initiated megestrol or other antihistamines were censored at the switching date to avoid overlapping exposure. For Sensitivity Analysis 2, time-at-risk began on the last administration date of the index medication and continued for up to 365 days, with the same censoring rules applied.

**Figure 1:**
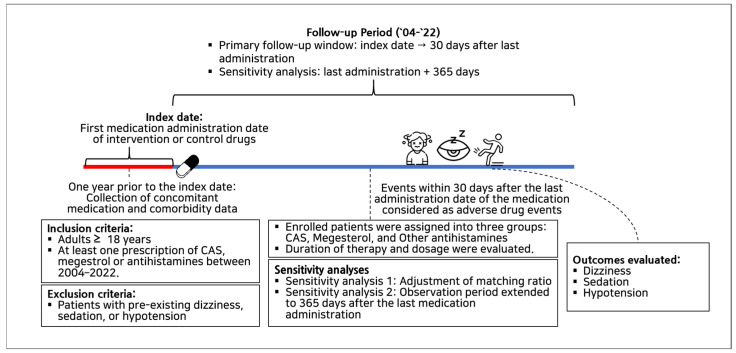
The study design. CAS; cyprogeptadine-based appetite stimulant.

The study design is illustrated in Figure 1.

### 2.3. Outcomes

The primary outcome variables were dizziness, sedation, and hypotension, defined as occurring if symptoms developed within 30 days of medication use. Concept sets for each outcome variable were defined including the corresponding source value, Korean Standard Classification of Diseases (KCD) codes, and concept_id. Detailed information regarding these concept sets is provided in Supplementary Tables S1 and S2, respectively.

### 2.4. Statistical Analysis

Patients in the CAS group were compared separately with each control group: megestrol (control group 1) and other antihistamines (control group 2). Specifically, in Study 1 CAS was compared to megestrol, while Study 2 compared CAS versus antihistamines. Propensity score matching [12] was applied using optimal matching methods with ratios of 1:3 for Study 1 and 1:5 for Study 2, respectively, based on concomitant medications, and laboratory parameters listed in Table 1. A standardized difference exceeding 0.1 was considered indicative of imbalance [13]. Survival analysis was performed using the Cox proportional hazards model. Statistical analysis was conducted using Python software, with descriptive data presented as mean ± standard deviation (SD), median (min–max), or as frequencies and percentages, as appropriate. Categorical variables were analyzed using chi-square or Fisher’s exact tests, while continuous variables were evaluated with *t*-tests. Univariate and multivariate Cox proportional hazards regression analyses were conducted to determine associations with outcome measures, with statistical significance defined at *p* < 0.05. Adjusted hazard ratio (aHR) were calculated after adjusting for age and sex. Python 3.12.4 was employed to conduct statistical analyses on data extracted from a PostgreSQL database.

### 2.5. Sensitivity and Subgroup Analyses

To evaluate the robustness of the findings, sensitivity analyses were conducted by adjusting the matching ratios and modifying the observation period to extend to 365 days after the last medication administration. Subgroup analyses were also conducted, stratifying patients by age (≥65 years vs. <65 years),medication duration (under 4 weeks, 4 weeks to 1 year, and over 1 year) and sex.

## 3. Results

### 3.1. Patient Characteristics

**Figure 2:**
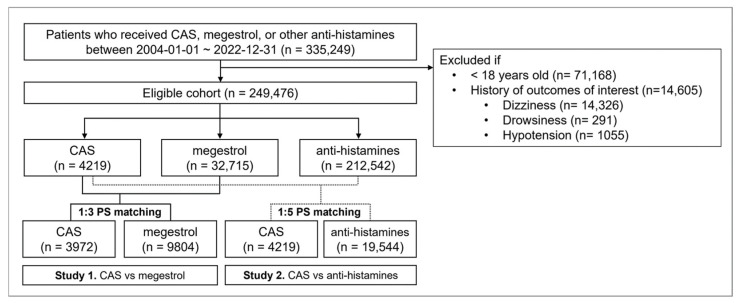
Study flow chart. CAS; cyprogeptadine-based appetite stimulant.

A total of 335,249 adult patients who received CAS, megestrol, or other antihistamines between 2004 and 2022 were initially identified. Following application of inclusion and exclusion criteria based on age and medical history, 249,476 patients were included in the final study cohort. Matched cohorts are illustrated in Figure 2. Although adults (≥18 years) were included according to the study criteria, the cohorts consisted predominantly of older adults. After matching, the mean age of CAS and control groups was approximately 70 years. Notably, 71.8% of CAS users were elderly individuals aged 65 or older. The treatment duration for CAS was longer compared to both megestrol and antihistamines. For CAS, the median treatment duration was 30 days (Q1: 8, Q3: 120), whereas for megestrol it was 32 days (Q1: 14, Q3: 89) and for antihistamines 15 days (Q1: 7, Q3: 47.5). The mean treatment duration was 178 days for CAS, 80 days for megestrol, and 73 days for antihistamines.

Prior to propensity score matching, the CAS group had a higher proportion of older female patients compared to both the megestrol and antihistamine groups, with significant baseline imbalances observed (STD > 0.1). Propensity score matching effectively balanced all measured covariates across the groups, with STD reduced to ≤0.1 after matching, thereby mitigating these initial disparities (Table 1 and Table 2).

### 3.2. Comparative Safety Profile of CAS and Megestrol

There were no significant differences in the risk of dizziness (aHR 1.02 [95% CI 0.70–1.50]), sedation (aHR 0.53 [95% CI 0.19–1.54]), or hypotension (aHR 0.70 [95% CI 0.34–1.44]) between the megestrol and CAS groups (Table 3a). These findings remained consistent across sensitivity analyses, including a 1:1 propensity score matching (sensitivity analysis 1) and an extended observation period of 365 days after the final drug administration for each group (sensitivity analysis 2) (Supplementary Tables S3 and S4, respectively).

### 3.3. Comparative Safety Profile of CAS and Antihistamines

In the initial analysis, CAS was associated with a modestly lower risk of dizziness compared with antihistamines (aHR 0.74 [95% CI 0.57–0.96]), whereas no significant differences were observed for sedation (aHR 1.05 [95% CI 0.46–2.38]) or hypotension (aHR 0.65 [95% CI 0.36–1.17]) (Table 3b). However, in the 1:1 matching sensitivity analysis, CAS was associated with a significantly lower risk of dizziness (aHR 0.56 [95% CI 0.41–0.78]) and hypotension (aHR 0.47 [95% CI 0.23–0.96]) compared to antihistamines, while the risk of sedation (aHR 0.67 [95% CI 0.23–1.93]) remained similar between the groups(Supplementary Table S3b). Additionally, in the extended 365-day observation window (sensitivity analysis 2), CAS remained associated with a significantly lower risk of dizziness (aHR 0.80 [95% CI 0.65–0.98]), with similar nonsignificant trends observed for sedation and hypotension (Supplementary Table S4).

### 3.4. Subgroup Analysis

Subgroup analysis based on the duration of use revealed no statistically significant differences as the duration increased. However, a significantly lower risk of dizziness was observed for CAS relative to both megestrol (aHR 0.38 [95% CI 0.19–0.76]) and antihistamines (aHR 0.61 [95% CI 0.41–0.92]). Similarly, CAS was associated with a markedly reduced risk of hypotension compared to megestrol (aHR 0.05 [95% CI 0.01–0.49]) and antihistamines (aHR 0.29 [95% CI 0.41–0.92]) (Table 4a).

Subgroup analysis by age did not reveal statistically significant differences between the CAS and control groups. When stratified by age, a higher risk of dizziness was observed in the ≥65 years age group relative to the <65 years age group within the antihistamine cohort (≥65 years, aHR 0.80 [95% CI 0.61–1.05]; <65 years, aHR 0.38 [95% CI 0.16–0.90]), although this difference did not reach statistical significance. The mean age of the CAS group after matching was 70 years. Limited data in the <65 years subgroup precluded conclusive analysis in certain comparisons (Table 4b). Sex-stratified subgroup analyses demonstrated no meaningful sex-specific differences in any of the outcomes (Table 4c). For example, in the CAS versus megestrol comparison, the aHR for dizziness in females was 1.12 [95% CI 0.47–2.52], with similar non-significant estimates observed for sedation and hypotension. Likewise, in the CAS versus antihistamine comparison, the female-specific aHR for dizziness was 0.52 [95% CI 0.28–0.91], and no sex-related differences were identified for sedation or hypotension. Overall, these analyses indicate that the comparative safety profile of CAS did not differ by sex.

## 4. Discussion

This retrospective study utilized the OMOP CDM to assess the safety profile of CAS compared to megestrol and other antihistamines. The findings indicate that CAS was not associated with increased risks of dizziness, sedation, or hypotension. Although the study population was defined as adults (aged ≥18 years), the distribution of age revealed that the CAS cohort was predominantly composed of older adults, reflecting real-world prescribing patterns in clinical nutrition practice. Therefore, while safety considerations are particularly relevant for older adults, the findings should be interpreted in the broader context of adult patients receiving CAS. The findings of this study support the use of CAS for appetite stimulation in adult patients, including those at older ages who may be more susceptible to antihistaminic effects, without additional short-term safety concerns relative to the comparators.

It should be acknowledged, however, that the clinical indications differed between exposures. While CAS is prescribed specifically for appetite stimulation at lower daily doses (6 mg/day) than those typically used for allergic conditions, whereas comparator antihistamines are primarily indicated for allergy at full therapeutic doses. Given that CAS is prescribed at a substantially lower daily dose (6 mg/day) than standard antihistaminic dosing, direct head-to-head safety comparisons with full-dose antihistamines should be interpreted with caution. A multicenter, randomized, double-blind, and placebo-controlled study [8] evaluated the efficacy and tolerability of CAS in poor appetite patients. Notably, even at a relatively low dose of 6 mg per day, which is well below the standard antihistaminic dose of 4 mg three times a day, CAS demonstrated significant weight gain and appetite improvement. This finding suggests that the reduced dosage may achieve orexigenic benefit while potentially minimizing dose-dependent adverse reactions such as somnolence and sedation, thereby supporting the tolerability of CAS.

Prior studies indicate that anticholinergic risks associated with CAS vary by dose and patient vulnerability, particularly among frail older adults. One study [14] investigated the prevalence of potentially inappropriate medication prescriptions among older and frail patient groups in U.S. nursing homes, revealing that the frequent prescriptions of cyproheptadine for rhinorrhea not as an appetite stimulant was inappropriate, as newer generations of antihistamines provide safer alternatives for rhinorrhea. Another study examining prescription patterns of anticholinergic agents in Korean elderly patients with dementia reported cyproheptadine as commonly prescribed to those with a high Anticholinergic Risk Scale score (3 points) [15,16]. These observations suggest that the anticholinergic risk may be influenced by dosage levels or the vulnerability of specific patient populations with comorbidities that heighten sensitivity to anticholinergic effects. In addition, cyproheptadine is associated with multiple clinically relevant drug–drug interactions and contraindications. As listed in product labeling [17], cyproheptadine may potentiate CNS depression when co-administered with other CNS depressants (e.g., benzodiazepines, opioids, sedating antidepressants) and may increase anticholinergic burden when combined with other anticholinergic agents. Contraindications include use with monoamine oxidase inhibitors, angle-closure glaucoma, symptomatic prostatic hypertrophy, urinary retention, pyloroduodenal obstruction, and use in newborns, breastfeeding mothers, or debilitated elderly patients. These pharmacologic considerations add context to the real-world safety evaluation presented here.

In this study, we compared CAS with megestrol and antihistamines using real-world data to provide a more patient-centered understanding of its safety profile. CAS showed a safety profile comparable to megestrol, aligning with prior work that found no significant differences between megestrol and other appetite-stimulating options such as mirtazapine [18,19]. In the primary comparison with megestrol, no statistically significant differences were observed, and the wide confidence intervals likely reflect limited power for detecting modest risk differences. To the best of our knowledge, this is the first study to directly compare the safety of CAS and megestrol.

Cyproheptadine is a first-generation H1 antagonist with central penetration and potential anticholinergic effects, particularly in elderly patients, due to its ability to readily cross the blood–brain barrier [20,21,22]. Despite these physicochemical properties, the findings of this study demonstrated that the incidence rates per 1000 person-years for dizziness, sedation, and hypotension were lower for CAS than for megestrol and were even lower relative to other antihistamines. It is important to note that the dosage of cyproheptadine used in CAS (6 mg/day) is lower (less than one third of the daily recommended dose) than that employed for its antihistaminic indication whereas the comparators were used at their full therapeutic doses. This dosage differential may have contributed to the observed safety profile and represents a limitation for direct head-to-head inference with antihistamines used at full doses. We addressed confounding using propensity score matching and conducted multiple sensitivity analyses, yet residual confounding by indication and dose may remain.

Because the pivotal clinical trial [8] excluded geriatric patients, real-world evidence is critical for adult patients, particularly for those of advanced age, in whom hepatic or renal impairment may alter antihistamine decomposition [23,24,25,26,27,28,29,30].

Moreover, this study provides critical insights into the therapeutic efficacy and safety of CAS as an appetite stimulant, addressing a significant gap in treatment options for a condition with limited alternatives and notable safety concerns. Anorexia is highly prevalent in older adults, affecting approximately 20% of individuals [31]. This high prevalence stems from a multifactorial etiology, including aging-related physiological changes and comorbid conditions. Despite its clinical importance, pharmacological options for treating anorexia are limited. Megestrol, a commonly used appetite stimulant, is associated with increased risks of thrombotic events and potential mortality, leading the Beers Criteria to recommend its avoidance in elderly patients [32]. Considering these limitations, CAS may represent as a viable pharmacotherapeutic alternatives. Although CAS was used over a longer duration (average of 178 days) compared to the relatively shorter treatment periods observed for megestrol and antihistamines, the sensitivity analyses addressing early outcome occurrences confirmed the robustness of the safety findings. Regarding efficacy, while the present study focused on safety endpoints, it is essential to recognize that the therapeutic effectiveness of CAS in stimulating appetite has been established in the previous publication [8]. Evaluating the safety outcomes of CAS and generating robust real-world evidence to support its use is both timely and essential. Such evidence could inform safer and more effective management strategies for anorexia in elderly populations, ultimately enhancing clinical decision-making and patient care.

Daytime dizziness and sedation are acute side effects that particularly occur after the initial dose. However, these symptoms tend to diminish as patients adapt to the medication over time [33,34]. In our study, the mean treatment duration was significantly longer in the CAS group compared to the control groups (178 days for CAS, 80 days for megestrol, and 73 days for antihistamines). Notably, patients who continued CAS after the initial adaptation did not experience a significant increase in adverse events. This finding, combined with the lower incidence rate of adverse outcomes in the CAS group, suggests that CAS has a favorable long-term safety profile.

To ensure the robustness of our results, our risk assessment evaluated the incidence of study outcomes within 30 days following the final drug administration. Sensitivity analyses, including extending the follow-up period to 365 days and adjusting the propensity score matching ratio, consistently demonstrated that the incidence of adverse events remained stable. These results reinforce that CAS can be safely administered over extended periods without a marked increase in risk. Given the absence of statistically meaningful differences between males and females in subgroup analyses, the comparative safety profile of CAS appears consistent across sex. This supports its potential for long-term use when managed appropriately.

Although the study was conducted at a single institution, potentially contributing to wider confidence intervals for certain subgroup analyses due to a limited sample size, a large EHR dataset transformed into the CDM was used, and matched cohorts were employed to minimize bias. Sensitivity analyses consistently demonstrated that CAS maintained a favorable safety profile across all assessments, including long-term safety even with extended use.

This study acknowledges certain constraints that may influence its interpretation. First, the retrospective design inherently limits the ability to control for all potential confounding variables. Additionally, the use of EHRs may lead to underreporting of minor adverse events, particularly for patients prescribed drugs in outpatient settings who might not report resolved acute adverse events during subsequent visits. Furthermore, given that megestrol is primarily indicated for cancer patients, baseline characteristics between groups may differ. The distribution of treatment duration was highly skewed, with a small proportion of long-term users contributing to a higher mean. Therefore, the median duration is more reflective of typical exposure. Given the heavy right-skew in treatment duration, the median rather than the mean more accurately represents typical CAS exposure. Moreover, outcome misclassification cannot be ruled out. Mild or transient dizziness or drowsiness may not be recorded in routine outpatient encounters, potentially leading to an underestimation of event rates. Although such misclassification may be non-differential, and therefore bias associations toward the null, differential misclassification is also possible. CAS is often prescribed in nutrition or geriatric settings, where documentation practices may differ from allergy clinics that more commonly prescribe antihistamines. Differences in coding behavior across specialties could influence hazard ratio estimates, and this limitation should be considered when interpreting the findings. To address these limitations, off-label drug use was included in the EHR data, and propensity score matching was applied to ensure balanced cohorts. To further mitigate potential misclassification, outcome was assessed across multiple observation windows, including the primary 30-day risk period and an extended 365-day sensitivity analysis. Additionally, age and duration stratified subgroup analyses were also performed, which allowed us to examine whether reporting patterns differed across clinically distinct subpopulations. Despite extensive propensity score matching and sensitivity analyses, residual confounding by indication, frailty, or dose cannot be fully excluded, and thus strong causal claims regarding comparative safety should be avoided. In addition, megestrol users may also represent a more clinically vulnerable population with higher baseline frailty and comorbidities, making direct comparisons difficult. Although this study was conducted using de-identified EHR data with IRB approval, and standard prespecified analytic methods such as propensity score matching were employed, the possibility of sponsor-related bias remains given that the manufacturer of CAS funded the study and employs several authors. These considerations should be considered when interpreting the findings.

While these limitations warrant caution in causal interpretation, the convergence of results across multiple analytical approaches strengthens confidence in the overall safety profile observed for CAS. Taken together, the comprehensive safety evaluation including age-stratified subgroup analyses in this study indicated no elevated risk associated with CAS compared to these comparator drugs. Therefore, while concerns regarding the use of CAS in elderly patients persist, CAS can be utilized safely in this population as an appetite stimulant, akin to megestrol and other antihistamines. Given that megestrol has been associated with thrombotic risks and antihistamines with anticholinergic effects, our data suggest a lower risk for certain outcomes, particularly dizziness and hypotension, in some analyses.

## 5. Conclusions

This study presents a thorough evaluation of the safety profile of CAS in adult patients, particularly in comparison to megestrol and other antihistamines. The results indicate that CAS has a comparable and acceptable safety profile. CAS can serve as an effective and safe option for treating anorexia in the elderly population, offering a viable alternative to existing medications.

## Figures and Tables

**Table 1:** Baseline characteristics in cyproheptadine-based appetite stimulants (CAS) and megestrol groups.

		Pre-Match			Post-Match		
		CAS (n = 4219)	Megestrol (n = 32,715)	STD	CAS (n = 3972)	Megestrol (n = 9804)	STD
Demo-graphic	Sex	1634 (38.7%)	18,221 (55.7%)	−0.3	1578 (39.7%)	4261 (43.5%)	−0.08
	Age	70.5 ± 13.3	66.2 ± 12.4	0.3	70.3 ± 13.3	69.8 ± 11.5	0.04
	Prescription count	337.7 ± 751.5	132.9 ± 192.9	0.4	251.8 ± 416.6	182.4 ± 292.1	0.1
Co-morbidities	Cerebrovascular	735 (17.4%)	3141 (9.6%)	−0.2	650 (16.4%)	1370 (14.0%)	−0.08
	Depression	564 (13.4%)	511 (1.6%)	−0.5	405 (10.2%)	433 (4.4%)	−0.2
	Diabetes Mellitus	193 (4.6%)	1146 (3.5%)	−0.05	177 (4.5%)	430 (4.4%)	−0.004
	Dyslipidemia	484 (11.5%)	1223 (3.7%)	−0.3	425 (10.7%)	803 (8.2%)	−0.1
	Hypertension	542 (12.8%)	2435 (7.4%)	−0.2	485 (12.2%)	1085 (11.1%)	−0.04
	Schizophrenia	63 (1.5%)	95 (0.3%)	−0.1	42 (1.1%)	65 (0.7%)	−0.04
	Anxiety	1654 (39.2%)	14,860 (45.4%)	0.1	1515 (38.1%)	3692 (37.7%)	−0.001
Co-medications	ACEi/ARB	945 (22.4%)	5621 (17.2%)	−0.1	872 (22.0%)	2110 (21.5%)	−0.01
	Beta-blocker	812 (19.2%)	6012 (18.4%)	−0.02	740 (18.6%)	1768 (18.0%)	−0.02
	Calcium channel blocker	1241 (29.4%)	9687 (29.6%)	0.004	1161 (29.2%)	2864 (29.2%)	−0.0004
	Other anti-hypertensives	36 (0.9%)	340 (1.0%)	0.02	32 (0.8%)	77 (0.8%)	−0.002
	Loop diuretic	660 (15.6%)	7007 (21.4%)	0.1	617 (15.5%)	1562 (15.9%)	0.01
	Other diuretics	917 (21.7%)	8552 (26.1%)	0.1	861 (21.7%)	2133 (21.8%)	0.002
	Metformin	532 (12.6%)	3111 (9.5%)	−0.1	502 (12.6%)	1214 (12.4%)	−0.008
	Sulfonylurea	301 (7.1%)	2548 (7.8%)	0.02	282 (7.1%)	724 (7.4%)	0.01
	DPP-4 inhibitor	379 (9.0%)	1768 (5.4%)	−0.1	353 (8.9%)	852 (8.7%)	−0.008
	SGLT-2 inhibitor	48 (1.1%)	127 (0.4%)	−0.09	46 (1.2%)	98 (1.0%)	−0.02
	GLP-1 agonist	2 (0.0%)	8 (0.0%)	−0.01	2 (0.1%)	6 (0.1%)	0.006
	Alpha-glucosidase inhibitor	26 (0.6%)	336 (1.0%)	0.05	26 (0.7%)	72 (0.7%)	0.009
	Meglitinides	13 (0.3%)	119 (0.4%)	0.01	11 (0.3%)	28 (0.3%)	0.002
	Insulin	485 (11.5%)	4066 (12.4%)	0.03	459 (11.6%)	1195 (12.2%)	0.02
	Erythropoietin stimulating agent	163 (3.9%)	917 (2.8%)	−0.06	135 (3.4%)	335 (3.4%)	0.001
	Iron supplement	87 (2.1%)	384 (1.2%)	−0.07	86 (2.2%)	208 (2.1%)	−0.003
	Anticonvulsant	195 (4.6%)	597 (1.8%)	−0.16	167 (4.2%)	317 (3.2%)	−0.06
	Antidepressant	793 (18.8%)	1610 (4.9%)	−0.4	649 (16.3%)	1070 (10.9%)	−0.2
	Antipsychotic	695 (16.5%)	2589 (7.9%)	−0.3	586 (14.8%)	1134 (11.6%)	−0.1
Laboratory findings	estimated GFR	80.8 ± 30.2	86.5 ± 31.3	−0.07	81.7 ± 30.0	76.1 ± 26.7	−0.05
	Iron saturation	29.4 ± 21.2	28.1 ± 22.6	0.1	29.3 ± 21.5	28.9 ± 21.5	0.02
	Vitamin B12	1067.2 ± 1299.9	1601.6 ± 7183.5	0.05	1087.6 ± 1358.7	1860.4 ± 10,951.9	−0.01
	Hemoglobin	11.7 ± 2.0	11.3 ± 1.9	−0.1	11.7 ± 2.0	11.2 ± 1.9	0.01
	Ferritin	574.4 ± 2005.6	661.3 ± 3595.8	0.01	598.7 ± 2101.3	637.7 ± 5630.9	−0.004
	Hba1c	6.2 ± 1.1	6.5 ± 1.3	0.4	6.2 ± 1.1	6.4 ± 1.2	0.07
	HDL	50.5 ± 16.9	46.4 ± 16.6	0.5	50.1 ± 16.8	49.8 ± 16.7	0.1
	Cortisol	15.9 ± 11.2	17.3 ± 24.5	0.07	16.0 ± 11.2	17.8 ± 29.6	0.02
	Cortisol30	20.6 ± 9.7	22.5 ± 10.7	0.09	20.6 ± 9.9	23.9 ± 11.6	0.03
	Cortisol90	10.7 ± 7.4	20.4 ± 11.5	−0.01	10.7 ± 7.4	20.5 ± 1.4	0.003
	Serum Na	138.3 ± 4.2	137.1 ± 4.4	−0.2	138.3 ± 4.2	137.7 ± 4.2	0.003
	Total cholesterol	161.1 ± 45.7	157.1 ± 47.7	−0.2	160.8 ± 46.0	156.0 ± 45.6	0.06
	Folate	12.7 ± 14.4	13.1 ± 56.2	0.1	12.6 ± 14.5	15.1 ± 84.4	0.07
	Mean blood pressure	107.4 ± 16.2	109.6 ± 16.0	0.1	107.2 ± 16.1	112.5 ± 16.7	0.02

ACEi; angiotensin-converting-enzyme inhibitor, ARB; angiotensin receptor blocker, CAS; cyproheptadine-based appetite stimulants, DPP-4; dipeptidyl peptidase-4, GFR; glomerular filtration rate, GLP-1; glucagon-like peptide-1, Hba1c; hemoglobin a1c, HDL; high-density lipoprotein, SGLT-2, sodium-glucose cotransporter 2, STD; standard-deviation.

**Table 2:** Baseline characteristics in cyproheptadine-based appetite stimulants (CAS) and antihistamine groups.

		Pre-Match			Post-Match		
		CAS (n = 4219)	Antihistamine (n = 212,542)	STD	CAS (n = 4219)	Antihistamine (n = 19,544)	STD
Demo-graphic	Sex	1634 (38.7%)	93,583 (44.0%)	−0.1	1634 (38.7%)	7848 (40.2%)	−0.03
	Age	70.5 ± 13.3	51.0 ± 17.7	1.2	70.5 ± 13.3	70.0 ± 12.6	0.03
	Prescription count	337.7 ± 751.5	141.4 ± 344.2	0.3	337.7 ± 751.5	298.1 ± 651.7	0.07
Co-morbidities	Cerebrovascular	735 (17.4%)	14,915 (7.0%)	−0.3	735 (17.4%)	3145 (16.1%)	−0.04
	Depression	564 (13.4%)	3240 (1.5%)	−0.5	564 (13.4%)	1739 (8.9%)	−0.2
	Diabetes Mellitus	193 (4.6%)	5218 (2.5%)	−0.1	193 (4.6%)	899 (4.6%)	0.001
	Dyslipidemia	484 (11.5%)	11,015 (5.2%)	−0.2	484 (11.5%)	2155 (11.0%)	−0.01
	Hypertension	542 (12.8%)	13,529 (6.4%)	−0.2	542 (12.8%)	2438 (12.5%)	−0.01
	Schizophrenia	63 (1.5%)	1329 (0.6%)	−0.08	63 (1.5%)	238 (1.2%)	−0.03
	Anxiety	1654 (39.2%)	39,080 (18.4%)	−0.5	1654 (39.2%)	7292 (37.3%)	−0.04
Co-medications	ACEi/ARB	945 (22.4%)	19,426 (9.1%)	−0.4	945 (22.4%)	4238 (21.7%)	−0.02
	Beta-blocker	812 (19.2%)	18,053 (8.5%)	−0.3	812 (19.2%)	3579 (18.3%)	−0.03
	Calcium channel blocker	1241 (29.4%)	23,347 (11.0%)	−0.5	1241 (29.4%)	5541 (28.4%)	−0.03
	Other anti-hypertensives	36 (0.9%)	1211 (0.6%)	−0.03	36 (0.9%)	164 (0.8%)	−0.001
	Loop diuretic	660 (15.6%)	11,635 (5.5%)	−0.3	660 (15.6%)	2894 (14.8%)	−0.03
	Other diuretics	917 (21.7%)	17,101 (8.0%)	−0.4	917 (21.7%)	4073 (20.8%)	−0.03
	Metformin	532 (12.6%)	8402 (4.0%)	−0.3	532 (12.6%)	2423 (12.4%)	−0.01
	Sulfonylurea	301 (7.1%)	6069 (2.9%)	−0.2	301 (7.1%)	1408 (7.2%)	0.003
	DPP-4 inhibitor	379 (9.0%)	4706 (2.2%)	−0.3	379 (9.0%)	1711 (8.8%)	−0.01
	SGLT-2 inhibitor	48 (1.1%)	511 (0.2%)	−0.1	48 (1.1%)	207 (1.1%)	−0.01
	GLP-1 agonist	2 (0.0%)	65 (0.0%)	−0.009	2 (0.0%)	11 (0.1%)	0.004
	Alpha-glucosidase inhibitor	26 (0.6%)	799 (0.4%)	−0.03	26 (0.6%)	117 (0.6%)	−0.003
	Meglitinides	13 (0.3%)	396 (0.2%)	−0.02	13 (0.3%)	63 (0.3%)	0.003
	Insulin	485 (11.5%)	7858 (3.7%)	−0.3	485 (11.5%)	2137 (10.9%)	−0.02
	Erythropoietin stimulating agent	163 (3.9%)	2853 (1.3%)	−0.2	163 (3.9%)	683 (3.5%)	−0.02
	Iron supplement	87 (2.1%)	437 (0.2%)	−0.2	87 (2.1%)	248 (1.3%)	−0.08
	Anticonvulsant	195 (4.6%)	3373 (1.6%)	−0.2	195 (4.6%)	804 (4.1%)	−0.02
	Antidepressant	793 (18.8%)	7556 (3.6%)	−0.5	793 (18.8%)	2905 (14.9%)	−0.1
	Antipsychotic	695 (16.5%)	10,372 (4.9%)	−0.4	695 (16.5%)	2587 (13.2%)	−0.1
Laboratory findings	estimated GFR	80.8 ± 30.2	89.0 ± 27.4	0.3	80.8 ± 30.2	79.2 ± 28.5	0.02
	Iron saturation	29.4 ± 21.2	30.0 ± 21.4	0.3	29.4 ± 21.2	30.4 ± 21.6	0.02
	Vitamin B12	1067.2 ± 1299.9	1167.5 ± 3306.7	0.3	1067.2 ± 1299.9	1377.2 ± 5345.6	0.05
	Hemoglobin	11.7 ± 2.0	13.2 ± 2.0	−0.02	11.7 ± 2.0	12.1 ± 2.0	−0.01
	Ferritin	574.4 ± 2005.6	521.6 ± 2790.1	0.08	574.4 ± 2005.6	586.3 ± 4616.3	0.001
	Hba1c	6.2 ± 1.1	6.2 ± 1.1	0.6	6.2 ± 1.1	6.3 ± 1.1	0.03
	HDL	50.5 ± 16.9	52.7 ± 15.6	0.4	50.5 ± 16.9	51.0 ± 16.0	0.02
	Cortisol	15.9 ± 11.2	14.2 ± 26.2	0.1	15.9 ± 11.2	15.4 ± 11.9	0.02
	Cortisol30	20.6 ± 9.7	20.2 ± 10.8	0.2	20.6 ± 9.7	21.4 ± 11.5	0.03
	Cortisol90	10.7 ± 7.4	18.6 ± 10.3	−0.03	10.7 ± 7.4	21.4 ± 6.7	−0.005
	Serum Na	138.3 ± 4.2	139.8 ± 3.0	0.5	138.3 ± 4.2	139.5 ± 3.5	0.004
	Total cholesterol	161.1 ± 45.7	178.1 ± 41.1	0.05	161.1 ± 45.7	163.3 ± 42.1	0.004
	Folate	12.7 ± 14.4	13.1 ± 26.0	0.3	12.7 ± 14.4	15.6 ± 43.9	0.08
	Mean blood pressure	107.4 ± 16.2	110.5 ± 16.5	0.4	107.4 ± 16.2	113.2 ± 17.0	0.02

ACEi; angiotensin-converting-enzyme inhibitor, ARB; angiotensin receptor blocker, CAS; cyproheptadine-based appetite stimulants, DPP-4; dipeptidyl peptidase-4, GFR; glomerular filtration rate, GLP-1; glucagon-like peptide-1, Hba1c; hemoglobin a1c, HDL; high-density lipoprotein, SGLT-2, sodium-glucose cotransporter 2, STD; standard-deviation.

**Table 3:** Results of the risk of adverse events of cyproheptadine-based appetite stimulants (CAS) compared to the control groups.

(**a**) CAS vs. megestrol					
**Outcomes**	**Patient-Year**	**Events**	**Rate per 1000 Patient-Years**	**aHR (95% CI)**	
Dizziness					
Megestrol	2436	75	30.8	1.02 (0.70–1.50)	
CAS	2172	63	29.0		
Sedation					
Megestrol	2452	15	6.1	0.53 (0.19–1.54)	
CAS	2197	8	3.6		
Hypotension					
Megestrol	2448	27	11.0	0.70 (0.34–1.44)	
CAS	2199	13	5.9		
aHR: age, sex adjusted.					
(**b**) CAS vs. antihistamines					
**Outcomes**	**Patient-Year**	**Events**	**Rate per 1000 Patient-Years**	**aHR (95% CI)**	
Dizziness					
Antihistamines	5412	303	56.0		0.74 (0.57–0.96)
CAS	2339	78	33.3		
Sedation					
Antihistamines	5480	24	4.4		1.05 (0.46–2.38)
CAS	2366	8	3.4		
Hypotension					
Antihistamines	5464	63	11.5		0.65 (0.36–1.17)
CAS	2368	14	5.9		
aHR: age, sex adjusted.					

**Table 4:** Results of subgroup analyses: (**a**) Duration of use; (**b**) Age; (**c**) Sex.

(**a**) Duration of use			
		**CAS vs. Megestrol**	**CAS vs. Other Antihistamines**
**Dizziness**			
Duration	<4 weeks	1.78 (0.89–3.55)	0.97 (0.55–1.70)
	4 weeks~1 year	1.35 (0.86–2.11)	0.79 (0.55–1.13)
	≥1 year	0.38 (0.19–0.76)	0.61 (0.41–0.92)
**Sedation**			
Duration	<4 weeks	0.75 (0.10–5.91)	1.00 (0.13–7.59)
	4 weeks~1 year	0.75 (0.21–2.63)	0.89 (0.27–2.98)
	≥1 year	0.22 (0.03–1.55)	1.28 (0.38–4.32)
**Hypotension**			
Duration	<4 weeks	1.47 (0.32–6.67)	0.79 (0.19–3.27)
	4 weeks~1 year	1.08 (0.50–2.34)	0.94 (0.47–1.91)
	≥1 year	0.05 (0.01–0.49)	0.29 (0.09–0.97)
CAS; cyproheptadine-based appetite stimulant			
(**b**) Age			
		**CAS vs. Megestrol**	**CAS vs. Other Antihistamines**
**Dizziness**			
Age	≥65 years old	1.22 (0.80–1.86)	0.80 (0.61–1.05)
	<65 years old	1.02 (0.70–1.50)	0.38 (0.16–0.90)
**Sedation**			
Age	≥65 years old	0.59 (0.20–1.79)	1.14 (0.49–2.64)
	<65 years old	0.53 (0.19–1.54)	-
**Hypotension**			
Age	≥65 years old	0.72 (0.34–1.52)	0.77 (0.42–1.42)
	<65 years old	0.70 (0.34–1.44)	-
CAS; cyproheptadine-based appetite stimulant			
(**c**) Sex			
		**CAS vs. Megestrol**	**CAS vs. Other Antihistamines**
**Dizziness**			
Sex	Female	1.12 (0.47–2.52)	0.52 (0.28–0.91)
**Sedation**			
Sex	Female	0.69 (0.14–2.58)	0.83 (0.22–2.30)
**Hypotension**			
Sex	Female	0.78 (0.19–2.17)	0.58 (0.23–1.36)
CAS; cyproheptadine-based appetite stimulant			

## Data Availability

The patient-level data supporting this study were derived from SNUH’s EHR system and formatted in a protected CDM. Data that can view all the records of a patient are difficult to share due to the policy of the Seoul National University Hospital. Upon reasonable request, aggregated statistical outputs produced on the secure server—reflecting the desired analyses—can be provided. Readers interested in these summary results should contact the corresponding author.

## Supplementary Materials

The following supporting information can be downloaded at: https://www.mdpi.com/article/10.3390/jcm15010054/s1, Supplementary Table S1. Definitions of drug concept sets and distribution of drug users by groups (S1a) Definitions of drug concept sets (S1b) Distribution of drug users by groups; Supplementary Table S2. Definitions of outcome concept sets; Supplementary Table S3. Results of sensitivity analysis 1 (1:1 matched analysis) (S3a) Cyproheptadine-based appetite stimulants (CAS) vs. megestrol (S3b) CAS vs. antihistamines; Supplementary Table S4. Results of sensitivity analysis 2 (Follow-up extended to 365 days after the last drug administration) (S4a) Cyproheptadine-based appetite stimulants (CAS) vs. megestrol (S4b) CAS vs. antihistamines.

## Abbreviations

The following abbreviations are used in this manuscript:

CAS	Cyproheptadine-based appetite stimulant
CDM	Common data model
aHR	Adjusted hazard ratio
