# Supplementary material for: Real-World Safety of Cyproheptadine-Based Appetite Stimulants: An Electronic Health Record-Based Retrospective Cohort Study in Adult Patients
Source: J Clin Med. 2025 Dec 21;15(1):54. doi: 10.3390/jcm15010054 (PMC12787220; doi:10.3390/jcm15010054)
Supplement: Supplementary file 1 [file jcm-15-00054-s001.zip › jcm-4006519-supplementary.pdf]

Supplementary Materials

Table S1. Definitions of drug concept sets and distribution of drug users by groups

(S1a) Definitions of drug concept sets

(S1b) Distribution of drug users by groups

Table S2. Definitions of outcome concept sets

Table S3. Results of sensitivity analysis 1 (1:1 matched analysis)

(S3a) Cyproheptadine-based appetite stimulants (CAS) vs. megestrol

(S3b) CAS vs. antihistamines

Table S4. Results of sensitivity analysis 2 (Follow-up extended to 365 days after the last drug administration)

(S4a) Cyproheptadine-based appetite stimulants (CAS) vs. megestrol

(S4b) CAS vs. antihistamines

Table S1. Definitions of drug concept sets and distribution of drug users by groups

(S1a) Definitions of drug concept sets

Source code	source_name	omop_concept_id	Group
AZL	Azelastine 1mg tab	42963874	antihistamine
CPH	Cyproheptadine 4mg tab	40166704	antihistamine
CRS	Cetirizine/Pseudoephedrine 5mg/120mg cap	42957281	antihistamine
CTR	Cetirizine 10mg tab	40228214	antihistamine
DLRT5	Desloratadine 5mg tab	19096758	antihistamine
DMH4CIN2	Dimenhydrinate/Cinnarizine 40mg/20mg tab	19107656	antihistamine
DMHD	Dimenhydrinate 50mg tab	19020367	antihistamine
DXLB6	Doxylamine succinate/Pyridoxine HCl 10mg/10mg DR tab	43526401	antihistamine
EBS	Ebastine 10mg tab	19064800	antihistamine
EBSS	Ebastine 1mg/ml syrup	19092435	antihistamine
FXFN	Fexofenadine 120mg tab	40223807	antihistamine
FXFN18	Fexofenadine 180mg tab	40223815	antihistamine
FXFN3	Fexofenadine 30mg tab	40223821	antihistamine
FXFN6PE	Fexofenadine/Pseudoephedrine 60mg/120mg tab	42957488	antihistamine
LCTR5	Levocetirizine 5mg tab	40163630	antihistamine
LCTRS	Levocetirizine 0.5mg/ml syrup	40163626	antihistamine
LRT	Loratadine 10mg tab	1107882	antihistamine
MQT	Mequitazine 5mg tab	19004269	antihistamine
MQTS	Mequitazine 0.5mg/ml syrup	19128969	antihistamine
MTL1LCT5	Montelukast(-Na)/Levocetirizine HCl 10mg/5mg cap	42964084	antihistamine
MTL5LCT5	Montelukast(-Na)/Levocetirizine HCl 5mg/5mg chew tab	2071193	antihistamine
MZLS	Mizolastine 10mg tab	19107209	antihistamine
PT	Pseudoephedrine/Triprolidine 60mg/2.5mg tab	40237822	antihistamine
RNEBS	Ebastine/Pseudoephedrine 10mg/120mg cap	42965082	antihistamine
TST	Trestan* cap	2001000036	trestan
MGS	Megestrol acetate 40mg tab	40164968	megestrol
MGSS	Megestrol 40mg/ml, 240ml suspension	21030160	megestrol
MGSS10	Megestrol 40mg/ml, 10ml pkg suspension	42920598	megestrol
MGSS20	Megestrol 40mg/ml, 20ml pkg suspension	42920254	megestrol
MGSS5	Megestrol 125mg/ml suspension pkg	1301001	megestrol
MGSS5P	Megestrol 125mg/mLsuspension pkg	1301001	megestrol

(S1b) Distribution of drug users by groups

Group	Drug name	Number of drug users
cyproheptadine-based appetite stimulants (CAS)	Trestan	4219
megesterol	Megestrol	32715
antihistamine	Ebastine	54594
antihistamine	Fexofenadine	48929
antihistamine	Levocetirizine	43450
antihistamine	Cetirizine	24680
antihistamine	Azelastine	18675
antihistamine	Triprolidine	9156
antihistamine	Dimenhydrinate	7156
antihistamine	Mequitazine	3102
antihistamine	Loratadine	2006
antihistamine	Doxylamine	649
antihistamine	Mizolastine	145

Table S2. Definitions of outcome concept sets

Outcome	Source_name	KCD7_id	OMOP concept_id
Dizziness	Difficulty maintaining balance		4097170
	dizziness		4223938
	dizziness, orthostatic		4012243
	drowsy consciousness		4152347
	drowsy mentality		4152347
	sleeping tendency		4152347
	vertigo		439383
	vertigo attack		4172609
	vertigo	R42	439383
	vertigo	R42	4172609
	benign paroxysmal positional vertigo	H811	81878
	dizziness	R42	4223938
	central vertigo	H814	381035
	peripheral vertigo	H813	78162
	peripheral vertigo, not otherwise specified	H813	78162
	somnolence	R400	4152347
	drowsiness	R400	4152347
	Delayed hydrops	H810	78162
	disorder of ligament	M242	4335745
	pathological dislocation and subluxation of joint. nec	M243	4335746
	osteonecrosis due to drugs	M871	4335745
	supervision of pregnancy with other poor reproductive or obstetric history	Z352	4250121
	Benign Paroxysmal Postural Vertigo	H811	81878
	Open fracture of great toe		439383
	peripheral vertigo	H8138	78162
	peripheral vertigo, not otherwise specified	H8138	78162
	Aural vertigo	H8130	78162
	Other peripheral vertigo	H8138	78162
	Vertigo from infrasound	T752	439383
	Vertiginous migraine	G438	4335745
	Delayed endolymphatic hydrops	H810	78162
	Migrainous vertigo	G433	4335745
	Benign recurrent vertigo	R42	4335746
	Dizziness and giddiness	R42	433316
	Dizziness, migrainous	R42	4335745
	Orthostatic dizziness	R42	4250121

	Cardiogenic vertigo	R42	439383
	Vestibular migraine	G438	4335745
	Benign paroxysmal positional vertigo, idiopathic	H811	81878
	Benign paroxysmal positional vertigo, secondary	H811	81878
	Central vertigo, vascular	H814	381035
	Central vertigo, degenerative	H814	381035
	Central vertigo, metabolic	H814	381035
	Central vertigo, tumor associated	H814	381035
	Single episodic vertigo, unspecified	R42	439383
Sedation	drowsy consciousness		4152347
	drowsy mentality		4152347
	hypersomnia		438134
	sleep attack		438134
	sleeping tendency		4152347
	sleeping, increased		438134
	hypersomnia	G471	438134
	narcolepsy	G474	436100
	primary hypersomnia	F511	4262584
	hypersomnia-related to other condition	F511	438134
	sleep disorder due to other condition, hypersomnia type	G471	438134
	recurrent hypersomnia	F511	443528
	recurrent hypersomnia	F511	438134
	idiopathic hypersomnia	F511	43531627
	idiopathic hypersomnia	F511	4262584
	posttraumatic hypersomnia	F511	438134
	subwakefulness syndrome	F518	438134
	somnolence	R400	4152347
	drowsiness	R400	4152347
	Idiopathic hypersomnia	G471	43531627
	Idiopathic hypersomnia	G471	438134
	Post traumatic hypersomnia	F511	438134
	Narcolepsy with cataplexy	G474	437854
	Narcolepsy without cataplexy	G474	43531721
	Narcolepsy without cataplexy	G474	436100
hypotension	blood pressure, decreased		317002
	blood pressure, low		317002
	hypotension		317002
	hypotension, orthostatic		319041
	spontaneous intracranial hypotension	G448	317002
	hypotension	I959	317002

orthostatic hypotension	I951	319041
low blood pressure	R031	317002
Other hypotension	I958	317002

OMOP: Observational Medical Outcomes Partnership

Table S3. Results of sensitivity analysis 1 (1:1 matched analysis)

(S3a) Cyproheptadine-based appetite stimulants (CAS) vs. megestrol

Outcomes	Patient-year	Events	Rate/1000 Patient-year	HR (95% CI)
Dizziness				
Megestrol	976	33	33.8	0.93 (0.58 – 1.49)
CAS	2172	63	29.0	
Sedation				
Megestrol	985	5	5.1	0.60 (0.16 – 2.24)
CAS	2197	8	3.6	
Hypotension				
Megestrol	985	12	12.2	0.65 (0.28 – 1.51)
CAS	2199	13	5.9	

CAS: cyproheptadine-based appetite stimulant, HR: hazard ratio

(S3b) CAS vs. antihistamines

Outcomes	Patient-yr	Events	Rate/1000 Patient-yr	HR (95% CI)
Dizziness				
Anti-histamines	1195	82	68.6	0.56 (0.41 – 0.78)
CAS	2339	78	33.3	
Drowsiness				
Anti-histamines	1210	7	5.8	0.67 (0.23 – 1.93)
CAS	2366	8	3.4	
Hypotension				
Anti-histamines	1198	18	15.0	0.47 (0.23 – 0.96)
CAS	2368	14	5.9	

CAS: cyproheptadine-based appetite stimulant, HR: hazard ratio

Table S4. Results of sensitivity analysis 2 (Follow-up extended to 365 days after the last drug administration)

(S4a) Cyproheptadine-based appetite stimulants (CAS) vs. megestrol

Outcomes	Patient-year	Events	Rate/1000 Patient-year	HR (95% CI)
Dizziness				
Megestrol	11329	157	13.9	1.03 (0.78 – 1.35)
CAS	5773	96	16.6	
Sedation				
Megestrol	11427	23	2.0	0.80 (0.37 – 1.75)
CAS	5835	12	2.1	
Hypotension				
Megestrol	11414	44	3.8	1.08 (0.64 – 1.81)
CAS	5833	24	4.1	

CAS: cyproheptadine-based appetite stimulant, HR: hazard ratio

(S4b) CAS vs. antihistamines

Outcomes	Patient-year	Events	Rate/1000 Patient-year	HR (95% CI)
Dizziness				
Anti-histamines	23103	498	21.6	0.80 (0.65 – 0.98)
CAS	6163	114	18.5	
Drowsiness				
Anti-histamines	23397	36	1.5	1.16 (0.60 – 2.27)
CAS	6230	12	1.9	
Hypotension				
Anti-histamines	23331	110	4.7	0.85 (0.55 – 1.31)
CAS	6228	26	4.2	

CAS: cyproheptadine-based appetite stimulant, HR: hazard ratio
